# Jojoba Seed Oil as Feed Additive for Sustainable Broiler Meat Production under Hot Climatic Conditions

**DOI:** 10.3390/ani12030273

**Published:** 2022-01-22

**Authors:** Ahmed A. A. Abdel-Wareth, Muhammad Mobashar, Anwar Shah, Abu Bakkar Sadiq

**Affiliations:** 1 Department of Animal and Poultry Production, Faculty of Agriculture, South Valley University, Qena 83523, Egypt; 2 Department of Animal Nutrition, The University of Agriculture, Peshawar 25120, Pakistan; 3 College of Veterinary Sciences, The University of Agriculture, Peshawar 25120, Pakistan; anwar.shah54@gmail.com (A.S.); bbkrsdq@gmail.com (A.B.S.)

**Keywords:** broiler, jojoba oil, meat physical trait, productive performance, meat quality

## Abstract

**Simple Summary:**

Sustainable poultry production has become essential to satisfy a growing global demand for high-quality meat protein. It is of the utmost importance to explore the merit of a new phytobiotic for the sustainable production of broiler chickens under tropical weather conditions. Thus, this study was conducted to evaluate supplementation of jojoba bioactive lipid compounds to broiler diets and its effects on growth performance and meat quality. The growth performance of broiler chickens fed with jojoba seed oil under tropical hot climatic conditions was linearly improved compared to the control group. Jojoba seed oil supplementation lowered abdominal fat and increased dressing percentage and meat quality compared to the control group.

**Abstract:**

This study aimed to evaluate the impact of dietary addition of jojoba seed oil on productive performance, physicochemical attributes and carcass quality of broiler meat under tropical weather conditions. A total of 384 one-day-old Ross-308 were subdivided into four dietary treatments of jojoba seed oil: 0, 50, 100 and 150 mg/kg of control diet. Each treatment group included twelve replicates with eight birds each. The results showed that dietary supplementation of jojoba seed oil linearly increased (*p* < 0.01) feed intake, body weight gain and improved (*p* < 0.01) feed conversion ratio. Interestingly, diets supplemented with jojoba seed oil linearly (*p* < 0.05) improved the percentage of dressing and reduced abdominal fat percentage compared to the control group. Dietary supplementation of jojoba seed oil showed no effects (*p* ≥ 0.05) on the weight of internal organs, including liver, heart, gizzard, spleen and pancreas of broiler chickens. Increasing jojoba seed oil levels in the diet decreased (*p* < 0.001) percentages of cook and drip losses of breast and leg (drumstick and thigh) muscles of broilers. It was concluded that jojoba seed oil used as a feed additive up to 150 mg/kg improves growth performance and meat quality of broiler chickens in tropical weather conditions.

## 1. Introduction

Controlling and understanding high environmental temperatures is crucial for successful broiler chicken production and meat quality. The hot environmental condition is one of the most important environmental stressors challenging broiler chicken production worldwide [1,2]. Reduced growth performance, low meat quality and less safety for broiler chickens are some of the harmful effects due to heat stress [1]. The hot environmental and nutritional factors are affecting the intensive broiler production, and broilers are very susceptible to high temperatures, heat anxiety in the summer season being correlated with reductions in growth performance in traditional houses, whose ventilation systems are not efficient at keeping the birds cool.

Nutrition is a key factor that mostly has an impact on feed efficiency, and hence production efficiency and meat quality of the poultry. High meat production and reduced mortality of poultry through natural growth promoters, as a substitute for antibiotic growth promoters (AGPs), are needed to ensure a decent nutritional strategy.

The rising concerns about antibiotic resistance [3,4], due to bacterial cross and multiple resistances [5], have resulted in an increased number of research studies on antibiotic alternatives, such as herbal plants and essential oils, as effective substitutes [6,7,8,9,10,11,12]. Essential oils have antimicrobial, hypercholesteremic, antioxidant and immune-stimulating properties [13] that could sustain the production and quality of poultry meat. Plant bioactive lipid compounds (PBLC), commonly known as essential oils [14], can be added to broiler diets with the aim to enhance their growth performance. PBLCs can be incorporated as feed additives for technological enhancement of feed safety and quality, as well as improved antioxidants, antimicrobials and digestive stimulants that can improve the growth performance and quality of poultry meat, mainly due to phenolic contents [13,15,16].

Jojoba plant (*Simmondsia chinensis* L.) is a perennial plant belonging to the Simmondsiaceae family and is cultivated in Egypt; Jojoba seed contains approximately 50% oil [17]. Jojoba seed oil is widely used in pharmaceutics and cosmetic formulation due to its unique structural characteristics and beneficial health and immunity enhancement effects [18]. It has antimicrobial and antioxidant properties [19,20] and hypocholesteremic effects [18,19,20], which, mainly due to their nutritional and therapeutic values, may result in better productive performance of broiler chickens.

Nevertheless, scarce literature is available on the influence of jojoba seed oil on production performance and meat quality of broilers. Therefore, this study was conducted to evaluate the efficacy of jojoba seed oil as a feed additive for sustainable broiler meat production in tropical weather conditions.

## 2. Materials and Methods

The protocol for this experiment was reviewed and approved by the South Valley University Institutional Animal Care and Use Committee (SVUAGR 432020).

### 2.1. Experimental Treatments

A total of 384 one-day-old Ross 308 were subdivided into four dietary treatments of jojoba seed oil: 0, 50, 100 and 150 mg/kg. Each group included twelve replicates with eight birds each. The experiment lasted for 5 weeks and consisted of two phases (0 to 21 days of age, starter phase; 22 to 35 days of age, grower phase). The diet was formulated to meet Ross 308 broiler nutrition recommendations. The birds were housed in a 120 × 70 × 50 cm^3^ (length, width and height, respectively) cage. The chickens were supplied with feed and water for *ad libitum* consumption during the experimental period. Each pen was equipped with hanging linear feeders and four nipple drinkers. The height of the nipple line was gradually raised as the birds grew. A lighting program of 24 h continuous light was applied. Broiler chicks were vaccinated with Newcastle Disease Infectious Bronchitis vaccine at the age of 7 days, followed by Infectious Bursal Disease vaccine (Gumboro) at the age of 12 days by means of eye drop. Feed formulation and composition of the experimental diets are presented in Table 1.

The birds during the study period had *ad libitum* access to water and feed. The average minimum and maximum outdoor temperatures were 34 and 40 °C (37 ± 2.2 °C) during the trial, and relative humidity was 30.2 and 36.5% (33.4 ± 2.3%), respectively. Brooding temperatures (indoor) were 38.3, 35.3 and 29.7 °C on 1–10, 11–21 and 22–35 days of age, respectively.

### 2.2. Jojoba Oil Preparation and Bioactive Components of Jojoba Oil Analysis

Jojoba seed oil was received from the Department of Pharmacognosy and Medicinal Plant, Faculty of Pharmacy, South Valley University. Jojoba seed oil was extracted by hydrodistillation in a Clevenger-type apparatus for 3 h at the Department of Medicinal and Aromatic Plants Research, National Research Center, Egypt, according to the Egyptian Pharmacopoeia [21]. Anhydrous sodium sulfate was used to dry jojoba seed oil. The oil was analyzed for its bioactive components using gas chromatography by a model Delsi 121C gas chromatograph (GC) fitted with a CPWA X 52 CB column (30 m × 0.32 mm; film thickness 0.25 μm). The oven temperature ranged from 50 to 300 °C at 4 °C/min. The temperatures 240 °C and 255 °C were programmed for injector and detector, respectively. Nitrogen was used as a carrier gas (flow rate of 1 mL min^−1^). Combined gas chromatography and mass spectrometry (GC/MS) were used for the identification of components using a Sigma 300 apparatus (PerkinElmer, Shelton, CT, USA) attached to an HP 5970 300 mass spectrometer (PerkinElmer, Shelton, CT, USA). Active components of jojoba seed oil are presented in Table 2.

### 2.3. Productive Performance

The average body weight (BW) and feed intake were recorded at the end of the experiment during both feeding phases [6,9]. Body weight and feed were measured on a weekly basis to calculate BW gain, feed intake and feed conversion ratio (FCR). The dead bird was calculated as the number of birds that died during the experimental period to the initial birds used. The magnitude of BW and feed intake were modified for bird mortality.

### 2.4. Carcass Parameters

At the end of the trial (35 days), 48 birds (4 birds per pen) per group were taken, based on the average final BW (2093 ± 26, g), and slaughtered by cutting the carotid artery, as described [6,9]. The hot carcass weight was recorded. The feet, feathers, head, internal organs, including liver, heart, gizzard, spleen, digestive system, and abdominal fat were removed from the plucked and eviscerated carcasses and weighted. The weight of the carcass was measured, and the percentage of dressing was calculated as follows:

$$\mathrm{Dressing}\%=\frac{\mathrm{Carcass}\;\mathrm{weight},g}{\mathrm{Live}\;\mathrm{BW},g}\times 100$$

### 2.5. Measurement of Meat Quality Attributes

The breast muscles and leg (drumstick and thigh) muscles from the left side of each bird were selected to record pH with a Knick digital pH meter (Broadly Corp. Santa Ana, CA, USA), cooking loss and drip loss, according to Korkeala et al. [22] and Nakamura and Katoh [23]. In order to determine the capability of meat to retain water, meat samples of about 2–2.5 cm of thickness and approximate weight of 100 g of leg or breast meat were inserted inside an expanded and closed bag and stored at 4 °C for 24 h. The meat of the leg or breast was weighed at the start and end of this time period, and the drip loss was expressed as the initial sample weight percentage. The cooking loss is a useful attribute to describe the water holding capacity of meat, which was measured on the meat samples used for drip loss. The samples of meat were weighed and water bathed until 70 °C temperature was reached. After cooling the samples at room temperature, they were weighed, and cooking loss was expressed as the initial raw weight of used samples.

### 2.6. Chemical Analysis

The dry matter (DM), gross energy (GE), crude protein (CP), crude fiber (CF), ether extract (EE), calcium (Ca) and phosphorus (P) chemical analysis of the used diets was conducted, as described by the Association of Official Analytical Communities (AOAC) [24].

### 2.7. Statistical Analysis

Results were evaluated using the PROC general linear models (GLM) system of statistical analysis software SAS 9.2 (SAS Institute, Inc.: Cary, NC, USA) [25]. The model includes the supplementation level of jojoba oil. The cage was the experimental unit for growth performance, and the animal was the experimental unit for carcass criteria and meat quality parameters. The linear and quadratic responses to the increasing levels of jojoba seed oil supplementations were determined by orthogonal polynomial contrasts. Duncan’s multiple range test was applied to compare treatment means. Significance was declared at *p* < 0.05.

## 3. Results

### 3.1. Feed Intake and Growth Performance

The effects of jojoba seed oil on feed intake and growth performance of broiler chickens under tropical weather conditions are summarized in Table 3 and Table 4. No mortalities were observed in the control group and the treatment groups. The results revealed that feed intake was linearly increased (*p* ≤ 0.001) with increasing jojoba seed oil. Supplementation of jojoba seed oil linearly improved (*p* ≤ 0.001) FCR from 21 to 35, and 1 to 35 days of age. Likewise, BW was linearly increased with the increasing level of dietary jojoba seed oil (*p* < 0.001) at 21 and 35 days of age. Accordingly, dietary jojoba seed oil (50, 100 or 150 mg/kg) increased BWG linearly (*p* < 0.01) compared to the control group throughout the experimental period (1–21, 22–35 and 1–35 days of age).

### 3.2. Dressing Percentage and Internal Organs

Effects of jojoba seed oil on carcass of broiler chickens at 35 days of age under tropical climatic conditions are presented in Table 5. The diets supplemented with jojoba oil linearly (*p* < 0.05) improved the dressing percentage and reduced abdominal fat percentage compared to the control group. On the other hand, supplementation of jojoba oil to broiler diets did not affect (*p* ≥ 0.05) the percentages of internal organs, including liver, heart, gizzard, spleen and pancreas.

### 3.3. Meat Physicochemical Properties

The meat physicochemical properties (Table 6) and pH values of the leg (drumstick and thigh) and breast muscles were not significantly affected by dietary jojoba seed oil supplementations under tropical weather conditions. The percentage of cook and drip losses of breast and leg muscles in birds fed with diets supplemented with jojoba seed oil at 50, 100 and 150 mg/kg, showed significant linear improvement (*p* < 0.01) compared to birds fed with the non-supplemented control diet during the experimental period (1–21, 22–35 and 1–35 days of age) under tropical climatic conditions.

## 4. Discussion

Due to the scarcity of available reports on the effect of jojoba seed oil on broiler chickens, a comparison was conducted with other studies that used other PBLC to sustain broiler production and meat quality. The beneficial effects of dietary inclusion of herbs on gut health, digestion of nutrients and intestinal integrity have been reported earlier [6,7].

The response of broilers to PBLC varied according to some factors, such as herbal chemical composition, levels and application methods, animal age and environmental conditions [6,7,8,9,10]. Varying results lead to difficulties in using herbal ingredients, as optimal dosage and mixture are yet to be identified. Thus, it was vital to define active components in the studied jojoba seed oil (Table 2) and PBLC before its use, which may provide useful information about its effect on broiler chickens in the future. The anti-inflammatory, antioxidant and antibacterial properties of jojoba seed oil are mainly due to its active components [18].

The controlling of hot climatic conditions in broiler house is decisive in the successful meat production of broiler chickens. The hot weather conditions serve as severe environmental stressors, which challenge the global production of broilers worldwide [1,2]. Reduced growth performance, low meat quality and less safety for broilers are some of the harmful effects due to heat stress [1]. Many studies on jojoba seed oil showed an important role in antimicrobial, antioxidant, anti-inflammatory, antifungal and anti-hyperglycemia activities [18,19], which is mainly due to the active components present in jojoba seed oil. These beneficial effects might be directly associated with improvements in broiler performance under hot climatic conditions.

In our study, supplementations of jojoba seed oil in the broiler diet significantly improved BW and BWG, proving that jojoba oil has an essential effect on the efficiency of digested feed into BW gain under hot climatic conditions. Interestingly, an improvement of FCR was observed in the jojoba oil-supplemented groups compared to the control group and, therefore, a higher BWG was observed in this study. The improved FCR can result in increased growth performance and meat quality of broilers raised under hot climatic condition. Jojoba oil has antimicrobial and antioxidant activities [18,26] that may support improvements in broiler performance in this study. Supplementation of *Hippophae rhamnoides* extract as a phytogenic feed additive in broiler diets increased growth performance in chickens, leading to a subsequent improved economic return [12]. Furthermore, jojoba seed oil improved feed intake and FCR, and this may be related to improved feed utilization efficiency [27]. It also appears in the current study that jojoba oil reduced the impact of hot climatic conditions on performance and viability of broiler chickens. Likewise, phytogenic feed additives can improve the resistance of broilers to hot climatic conditions [28]. Ma et al. [29] reported that broiler chickens exposed to hot environmental temperatures had a lower average daily gain, breast muscle mass and muscle yield and increased feed-to-gain ratio. Inclusion of *Artemisia annua* at 1 g/kg in broiler diet could alleviate heat-stress-induced compromised growth performance and intestinal damage to broilers [28]. Moreover, jojoba seed oil led to an increase in bird feed intake, BW and BWG under tropical climatic conditions, due to its active components in a concentration-dependent manner. The effect of jojoba seed oil in increasing BWG may be due to stimulating the digestive system function and improving feed efficiency [27,30].

Furthermore, in our study, the linear effects found for the BWG and FCR during the experimental periods of feeding indicate that the supplementation of jojoba seed oil up to 150 mg/kg was still below the maximum dose to gain perceivable feed additive impact on the growth performance of broiler chickens under hot climatic conditions.

Concerning the carcass criteria in the current study, jojoba seed oil improved dressing percentage and decreased abdominal fat percentage at 35 days of age, which could be due to the biologically active chemical components of jojoba seed oil (Table 2). The decrease in abdominal fat is mainly due to PLBC in broilers’ diet improving activities of amylase and trypsin [31,32] and secretion of bile acids [18,26,33,34]. Unfortunately, no literature is available on the effect of jojoba seed oil on the carcass criteria of broilers; therefore, the comparisons were performed with other phytogenics. The current results of our study were consistent with the results reported by Khempaka et al. [35], in which the addition of dried *Mesembryanthemum cordifolium* at 0.5, 1.0, 1.5 and 2.0% had no significant effects on carcass characteristics and organs, but it decreased abdominal fat of broilers at 42 days of age. However, feeding dried pennyroyal at 5 g/kg resulted in positive effects on carcass traits in broilers at 42 days of age [36]. Moreover, Gurbuz and Ismael [37] reported that feeding 15 g/kg of dry peppermint leaves had not significantly affected the relative weight of liver, carcass yield or abdominal fat. The carcass characteristics, gizzard, intestine and abdominal fat of broiler chicks were also not affected by peppermint powder [38]. On the other hand, Nanekarani et al. [39] showed that abdominal fat in broilers decreased with the supplementation of 3.0 g/kg peppermint leaves. In addition, Al-Kassie [40] recorded positive effects in the carcass and liver yields of broilers due to the inclusion of dry peppermint powder at levels ranging from 2.5 to 15 g/kg. However, the results of supplementation of these phytogenics in broiler diets are still controversial among these studies, which could be due to the differences between various species and their compositions, as well as animal housing condition and health stage.

Broiler nutrition has an important impact on meat quality and safety. The effects of jojoba seed oil on broiler physiochemical meat quality have not been previously reported. In the current study, increasing jojoba seed oil levels in broiler diets significantly decreased cook loss and drip loss percentages of leg and breast muscles at 35 days of age under hot climatic conditions. These results indicate the addition of jojoba oil in broiler diets is able to improve breast meat cook loss. These positive impacts in cook loss and drip loss may be due to the antioxidant activity of jojoba seed oil, which might also have enhanced the oxidative stability and protective effects on broiler meat [17,18,26,41,42]. Furthermore, the addition of *Artemisia annua* at 1.0 g/kg to broiler diet improved the meat quality and the oxidative stability of breast and thigh muscles [43]. The improvements in cook loss may be related to the antioxidant activity of active components presented in PBLC, which might also have protective effects on chicks’ meat and enhance the oxidative stability of meat [44].

Considering the above variables, supplementation of broiler diets with jojoba oil up to 150 mg/kg improved meat production and meat quality of broilers under tropical climatic conditions. Furthermore, due to the fact that the report on the supplementation of jojoba seed oil in broilers is not investigated, jojoba seed oil could be served as feed additive to improve feed intake, FCR, BWG, abdominal fat and physicochemical meat quality under tropical hot conditions. Furthermore, hot environmental conditions impact gut resilience, and supplementation of jojoba seed oil may be used in broiler nutrition with the aim to effectively simultaneously maintain gut resilience induced by heat stress and consequently increase chicken performance. Further investigations are needed to compare the impact of jojoba seed oil on broiler productions under normal cold and hot conditions.

## 5. Conclusions

In conclusion, jojoba seed oil 150 mg/kg supplementation improved broiler growth performance and physiochemical meat quality under a hot climatic setting. Further studies to elucidate the mechanism of action and optimum supplementation levels of jojoba seed oil feed additive and its effects on physiological and nutritional response of chickens under different conditions need to be conducted.

## Figures and Tables

**Table 1:** Feed composition of the experimental diets.

Ingredients, g/kg	Starter Diet 1–21 Days	Grower Diet 22–35 Days
Corn	276	300
Sorghum	274	300
Soybean meal (CP 44%)	285	250
Corn gluten meal (CP 60%)	95.0	60.0
Premix ^a^ (minerals-vitamins)	3.00	3.00
Sunflower oil	30.0	55.2
Limestone	9.99	10.00
Dicalcium phosphate	19.9	17.9
Salt	3.90	3.90
L-lysine HCl	1.10	---
DL-methionine	0.41	---
Total	1000	1000
Analyzed chemical composition (%) ^b^		
DM	93.0	92.7
CP	23.2	21.3
EE	5.40	5.80
CF	2.60	3.80
Ash	6.70	6.20
Ca	1.31	1.30
P	0.72	0.71
Gross energy, Kcal/kg	4457	4608

^a^ Supplied mineral-vitamin premix contains per kg of feed: 80 mg cobalt; 400 mg iodine; 60 mg selenium; 1.200 mg iron; 2.000 mg copper; 18.000 mg manganese; 14.000 mg zinc; 1000.000 IU vitamin D; 2400.000 IU vitamin A; 800 mg vitamin K; 650 mg vitamin B1; 16.000 IU vitamin E; 1.600 mg vitamin B2; 1.000 mg vitamin B6; 6 mg vitamin B12; 400 mg folic acid; 8.000 mg niacin; 3.000 mg pantothenic acid; 40 mg biotin. ^b^ DM = dry matter, CP = crude protein, EE = ether extract, CF = crude fiber, Ca = calcium, P = phosphorus.

**Table 2:** The active components of jojoba seed oil.

No	Real Time (min)	Active Component Name	Concentration %
1	8.96	2-butyloctanol	0.42
2	10.39	Tetradecane,2,6,10-Trimethyl	0.27
3	10.75	1-Decanol-2,2dimethyl	0.44
4	12.04	Cis-9-Hexadecenoic acid	0.24
5	12.35	Isomyristic acid	0.37
6	13.91	Dodecanedioic acid	9.76
7	14.37	a-Bisabolol	1.15
8	14.76	Vitexin	0.87
9	15.16	3-Hydroxy-7,8,2trimethoxyfiavone	2.08
10	15.29	Elaidic-acid	2.71
11	15.6	1.15 pentadecanediol	18.53
12	15.82	Cis-10-Nonadecenoic acid	1.2
13	16.24	22-Tricosenaic acid	3.29
14	16.51	Linoleic acid	5.21
15	17.38	9-Hexadecen-I-al,(2)	11.95
16	18.75	2-10-Octadecen-1-olacetate	7.58
17	20.13	Epoxyaleic acid	1.46
18	20.9	Tetrahydrospirilloxamhin	2.47
19	21.7	Trans-13-Octadecenoic acid	11.8
20	21.9	Ouabagenin	18.29

**Table 3:** Effects of jojoba seed oil on body weight and body weight gain of broilers.

Items	Body Weight, g			Body Weight Gain, g		
	1 Day	21 Day	35 Day	1–21 Day	21–35 Day	1–35 Day
Jojoba seed oil levels, mg/kg						
0	44.3	676 ^b^	1994 ^c^	614 ^b^	1336 ^b^	1950 ^b^
50	43.5	711 ^a^	2092 ^b^	668 ^a^	1381 ^a^	2049 ^b^
100	44.4	713 ^a^	2125 ^a^	669 ^a^	1412 ^a^	2080 ^b^
150	44.3	726 ^a^	2162 ^a^	682 ^a^	1436 ^a^	2118 ^b^
SEM	0.65	11.5	26.5	16.3	18.4	23.7
*p*-value						
Linear	0.419	0.001	0.001	0.011	0.006	0.001
Quadratic	0.247	0.055	0.117	0.055	0.219	0.117

Means not sharing a common letter (^a–c^) in a column are significantly different (*p* < 0.05). SEM: standard error of the means (*n* = 12).

**Table 4:** Effects of jojoba seed oil on feed intake and FCR of broiler chickens.

Items	Feed Intake, g			Feed Conversion Ratio		
	1–21 Days	21–35 Days	1–35 Days	1–21 Days	21–35 Days	1–35 Days
Jojoba oil levels, mg/kg						
0	1025	2278 ^b^	3303 ^b^	1.670 ^a^	1.728 ^a^	1.715 ^a^
50	1041	2292 ^b^	3344 ^a^	1.560 ^b^	1.660 ^b^	1.632 ^b^
100	1030	2330 ^a^	3359 ^a^	1.540 ^b^	1.650 ^b^	1.615 ^b^
150	1029	2341 ^a^	3370 ^a^	1.510 ^b^	1.630 ^b^	1.591 ^b^
SEM	11.7	29.9	32.9	0.072	0.059	0.043
*p*-value						
Linear	0.135	0.004	0.041	0.027	0.011	0.002
Quadratic	0.361	0.978	0.314	0.079	0.057	0.159

Means not sharing a common letter (^a,b^) in a column are significantly different (*p* < 0.05). SEM: standard error of the means (*n* = 12).

**Table 5:** Effects of jojoba seed oil on carcass and internal organs of broiler chickens.

Items			Carcass and Internal Organs, %					
	Dressing	Fat	Liver	Heart	Gizzard	Spleen	Pancreas	Small Intestine
Jojoba seed oil levels, mg/kg								
0	75.28 ^c^	1.56 ^a^	1.98	0.48	1.70	0.17	0.26	2.54 ^b^
50	76.90 ^b^	1.08 ^b^	2.01	0.46	1.76	0.16	0.27	2.69 ^a^
100	77.61 ^a^	1.01 ^b^	2.06	0.43	1.78	0.15	0.28	2.73 ^a^
150	77.75 ^a^	0.99 ^b^	2.04	0.44	1.83	0.16	0.26	2.74 ^a^
SEM	1.01	0.025	0.179	0.069	0.022	0.099	0.011	0.098
*p*-value								
Linear	0.015	0.001	0.315	0.776	0.461	0.067	0.723	0.042
Quadratic	0.736	0.624	0.606	0.409	0806	0.045	0.643	0.643

Means not sharing a common letter (^a–c^) in a column are significantly different (*p* < 0.05). SEM: standard error of the means (*n* = 48).

**Table 6:** Effects of jojoba seed oil on physicochemical properties of broiler chickens.

Items	Breast Muscle			Leg Muscle		
	pH Value	Drip Loss, %	Cook Loss, %	pH Value	Drip Loss, %	Cook Loss, %
Jojoba seed oil levels, mg/kg						
0	5.77	21.76 ^a^	23.20 ^a^	5.69	20.98	24.13 ^a^
50	5.76	19.16 ^b^	21.49 ^b^	5.71	20.18	22.32 ^b^
100	5.81	18.99 ^b^	20.35 ^b^	5.73	20.07	21.66 ^b^
150	5.79	18.67 ^b^	20.15 ^b^	5.70	20.02	21.32 ^b^
SEM	0.066	0.47	1.15	0.076	0.68	0.72
*p*-value						
Linear	0.673	0.033	0.031	0.346	0.052	0.001
Quadratic	0.070	0.327	0.507	0.693	0.111	0.126

Means not sharing a common letter (^a,b^) in a column are significantly different (*p* < 0.05). SEM: standard error of the means (*n* = 48).

## Data Availability

Data presented in this study are available on fair request from the respective author.
